# The nature of allometry in an exaggerated trait: The postocular flange in *Platyneuromus* Weele (Insecta: Megaloptera)

**DOI:** 10.1371/journal.pone.0172388

**Published:** 2017-02-17

**Authors:** Andrés Ramírez-Ponce, Gabriela Garfias-Lozano, Atilano Contreras-Ramos

**Affiliations:** 1 Catedrático CONACYT, Laboratorio Regional de Biodiversidad y Cultivo de Tejidos Vegetales, Instituto de Biología, UNAM, Santa Cruz Tlaxcala, Tlaxcala, Mexico; 2 Recent graduate, Licenciatura en Biología, Facultad de Ciencias, UNAM, Mexico City, Mexico; 3 Instituto de Biología, UNAM, Departamento de Zoología, Mexico City, Mexico; Seconda Universita degli Studi di Napoli, ITALY

## Abstract

The origin and function of exaggerated traits exhibited by a great number of species with sexual dimorphism remain largely unexplored. The usual model considered as the evolutionary mechanism for the development of these structures is sexual selection. The nature of growth of the postocular flange (POF) in three species of the dobsonfly genus *Platyneuromus* (Megaloptera, Corydalidae, Corydalinae) is analyzed to explore sexual size dimorphism and allometric scaling. Results involve positive allometry of POF in males of two species, and negative allometry in males of one species, in general with a female-biased sexual dimorphism. We suggest an ancestral condition of dual incipient ornamentation in *Platyneuromus*, with a subsequent departure of size and shape of POF in males, triggered by sexual selection. Different sexual selection intensities may explain the parallel or divergent growth of POF within the scheme of dual ornamentation. Empirical behavioral data as well as a phylogenetic framework are necessary to clarify possible causes of phenotypic development, time of origin, and evolution of the POF.

## Introduction

The occurrence of different phenotypes among sexes, species, and supraspecific taxa has been traditionally a crucial aspect for the study of the origin and evolution of biodiversity, and much of this phenotypic diversity is the result of differential growth or allometry [1]: the study of how the change of traits or processes scale with size [2], as well as the causal mechanisms and their interpretation in terms of ecology and evolution. Allometry has been a classical quantitative approach for the evolutionary study of differential size relationships between different body parts [3] in several animal groups, vertebrates and invertebrates [4–10], dealing with diverse phenomena such as locomotion, thermoregulation, defense, species recognition, and others [11,12].

Allometry is a useful framework to study the ontogenetic basis of sexual dimorphism as growth trajectories that represent the processes that shape the organismal growth [13], and may be addressed at different biological levels of development [8], during growth (ontogenetic allometry), among individuals at the same developmental stage (static allometry), or among species (evolutionary allometry). Among these levels, alternative developmental strategies may arise: parallel growth trajectories during early differentiation of sexes, or divergent growth trajectories in a gradual differentiation of sexes [13]. In insects, there may be a high correlation between ontogenetic and static allometry, because the patterns of static allometry are established during larval development [14,15].

In many cases, the differential growth is markedly disproportionate, manifested in exaggerated structures in some species [12]. Sexually dimorphic traits, evident as disproportionate structures, are some of the most striking examples [13,16]. Another phenomenon related to sexual size dimorphism (SSD) and allometry that exhibits an interspecific pattern is Rensch´s rule, which predicts that when males are larger than females, the SSD increases with body size and when females are the largest sex, the SSD decreases in larger species [17,18], with sexual selection as the force that selects large male sizes [19]. However, in most ectotherm animals like insects, females are larger than males [18,20], producing a female-biased SSD.

Experimental and analytical evidence point out that sexual selection may be an important force that favors both sexual dimorphism and positive allometry in structures that often involve exaggerated traits [6,21–26], because of differential resource allocation in structures that favor mating success [23,27]. Yet, causes that turn these traits decoupled from the rest of the body to grow faster, larger [12,22] or with an increase in complexity are not always related to sexual selection. It has been demonstrated in several animal groups that an increase in ornament’s complexity may be a by-product of body size increase through time within clades [28,29].

The functional aspect of the exaggerated structure under sexual selection can arise as a weapon used in male to male combat or as a male ornament attractive to females [7,15,18], however, although both functional structures have derived from competition for reproduction, the nature and intensity of the selective forces within male to male competition or female mate choice differ, affecting the evolution of these traits [25,30].

Insects represent an evolutionary model for the study of allometry because they may take shape to the extreme, yielding some of the strangest-looking animals [26], as in stalked-eyed flies [31], butterflies [32], and beetles [10,33–37]. Another example is the order Megaloptera, which has some of the most impressive developments of cephalic structures [25]: mandible hypertrophy [38], and expansion of cephalic sclerites [39].

The dobsonfly genus *Platyneuromus* (Corydalidae, Corydalinae) is endemic to Mexico and Central America (northeastern Mexico to Panama); together with *Chloronia* and *Corydalus* conforms the New World lineage of dobsonflies [38,40]. It is distinguished by the presence of a postocular flange that can grow disproportionately in larger males [41], but females also develop it on a smaller scale. It possibly represents a sexually dimorphic trait analogous to the mandibles that undergo allometry in the dobsonfly genera *Corydalus* and *Acanthacorydalis* [38,39,41], but the nature, the causal mechanism, and the sexual meaning of this structure remains largely unexplored.

In the present study, we analyze the static allometry of the postocular flange for the three known species of *Platyneuromus* through linear and geometric morphometrics, in order to test differential patterns of intra and interspecific morphological variation. We use linear morphometry to analyze the scaling relationship of size change between the POF and standard body measures, and a geometric morphometric approach to analyze the correlation between size and form of the POF. We describe in-depth for the first time in the Megaloptera the phenomenon of allometry, including sexual size dimorphism (SSD) and the scaling of the postocular flange, aiming to elucidate evolutionary forces responsible for the allometry pattern.

## Material and methods

### Specimens

**Fig 1:**
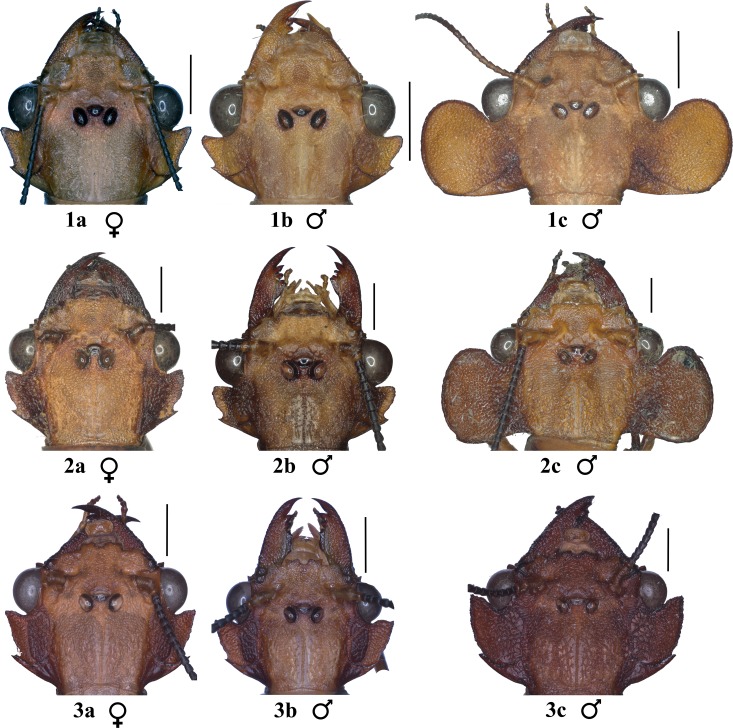
Head of *Platyneuromus* spp. 1a-c) *P*. *honduranus*, 2a-c) *P*. *soror*, 3a-c) *P*. *reflexus*.

Photographs of a total of 200 specimens of the three species of the genus *Platyneuromus* (Fig 1) were taken with a Carl Zeiss Axio Zoom V16 stereomicroscope: *P*. *honduranus* Navás (57 males, 54 females), *P*. *reflexus* Glorioso and Flint (6 males, 17 females), and *P*. *soror* (Hagen) (28 males, 38 females). All specimens were obtained from the National Collection of Insects, Instituto de Biología, UNAM, Mexico City. *P*. *honduranus* is known from southern Mexico through Guatemala and northwestern Honduras, *P*. *reflexus* is restricted to Chiapas (Mexico) and adjacent Guatemala, while *P*. *soror* is the most widespread species, ranging from northeastern Mexico, south into Costa Rica and northern Panama [39].

### Data acquisition (Fig 2)

To standardize the measuring procedure, a protocol for recording measurements was designed using a configuration of six landmarks with a comb and a fan made with MakeFan8 (http://www3.canisius.edu/~sheets/IMP%208.htm) following the morphological terminology of Glorioso [41]. The comb of six lines was designed from the joining of the eye and the POF (landmark 2), down to the posterior edge in straight vertical line; the fan was formed by 20 lines beginning at landmark 2, towards the base (landmark 6) and the apex (landmark 5) of the postocular spine. A) Linear morphometry. Three indicatives of standard morphological measurements (SMM) were collected [interantennal distance (IAD, head width across antennae), interocular distance (IOD, head width across eyes) and anterior wing length (AWL, right forewing length)], and three of the postocular flange (POF) [mesial width (MW, flange width at the middle; third line of the comb, from inner limit of the postocular plane to outer edge), diagonal length (DL, from the inner basal angle to the upper most distal point; eighth line of the fan), postocular spine length (SL, from the inner basal angle to the apex of the spine)]. The linear measurements were obtained with the software CoordGen8 using the Traditional Morphometrics Data Set Generator tool [42], with exception of the forewing length, which was obtained with a manual Vernier caliper (**S1 Table**). B) Geometric morphometry. The six landmarks were digitized from the POF in each picture using tpsDig2 ver. 2.22 [43], and the tps file was made and organized with the tpsUtility program ver. 1.65 [43].

**Fig 2:**
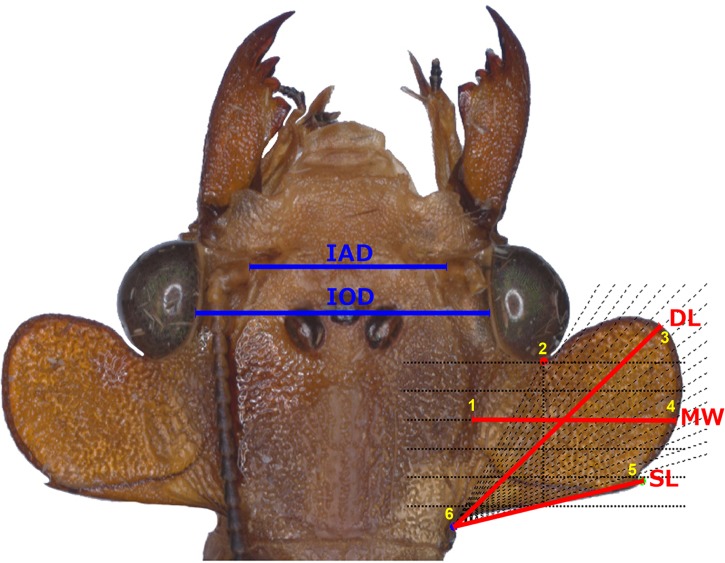
Measures and landmarks. a) Measures: BODY: interantennal distance (IAD), interocular distance (IOD), anterior wing length (AWL); POSTOCULAR FLANGE: mesial width (MW), diagonal length (DL), and postocular spine length (SL). b) Landmarks in yellow.

### Morphometric analysis

Mathematically, the rate of growth of two traits is described with the equation of the standard linear regression, and the slope denotes the allometric relation between the traits [1]. This was the conceptual and methodological framework for both, linear and geometric morphometric analyses. For all analyses, data were log transformed [44,45] in PAST [46]. Linear morphometry: Principal Component Analysis (PCA) was conducted with all traits as an exploratory test of the maximum amount of variance with the fewer uncorrelated variables to display interspecific data structure; Analysis of Pairwise Correlation incorporated all measurements pairs to test differential correlation patterns among the two types of traits, indicatives of body measures and of POF, in order to select the best independent body measure; Linear Regressions between interantennal distance and mesial width of the POF were calculated to test the allometric growth of the POF, and Analysis of Variance of all traits by species and sex were made to show sexual size dimorphism in the body and POF measures. All analyses were done with the software JMP [47]. Differences were considered significant at p < 0.05. Geometric morphometry: To explore the relation of variation between shape and size of the POF, shape variables of the Procrustes coordinates were analyzed through a regression as dependent variables, on the log of centroid size as independent variable, using MorphoJ 1.06d [48], with a permutation test against the null hypothesis of independence with 10,000 rounds for testing statistical significance [49]. The shape dependent change in size that represents the extremes for all species is illustrated by the transformation grid for the largest (male) and the smallest (female) specimens.

## Results

PCA, multivariate correlations, and logarithmic regression reveal clear intra and interspecific differences in morphological variation by distinct spatial distribution of the data, as well as different patterns of sexual dimorphism and allometric growth rates.

### Principal component analysis (Fig 3)

PCA for all traits shows high values of explained variance of the total shape variation, with 96% of the accumulated explained variance in only the first two principal components. In the first principal component (83.5%; p < 0.0001), the variables that most contribute to the variance of the data are DIO (0.433) and DL (0.432), while in the second principal component (13.1%; p<0.0001) is MW (0.719) and AWL (-0.540). The distribution of data shows a different pattern in the visual display of the data configuration and variation for each species.

**Fig 3:**
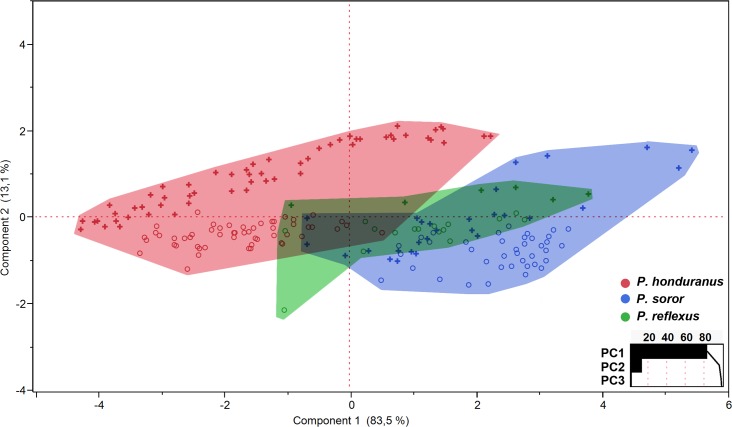
PCA of the three *Platyneuromus* species. Measurements of body and postocular flange are included for both sexes.

### Pairwise correlation

**Fig 4:**
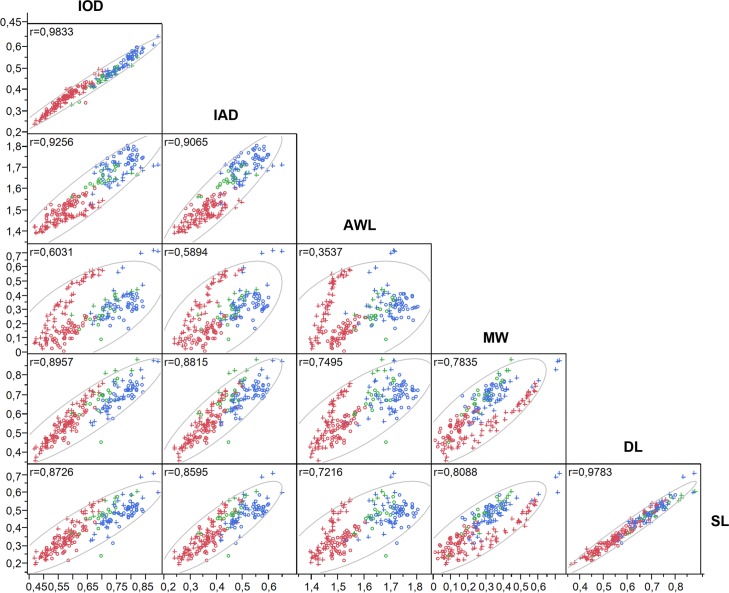
Analysis of pairwise correlation. Body measures are IAD, IOD, and AWL; postocular flange measures are MW, DL, and SL.

This analysis shows greatest values between the same type of traits, which exhibit, in some cases, a proportional growth close to a one-to-one correlation (range between SMM traits = 0.9833–0.9065; range between POF traits = 0.9783–0.7835). In contrast, when POF measures are correlated with SMM, the lowest correlations are obtained (range = 0.8957–0.3537), an indicative of different growth rates between the two types of traits (Fig 4). All tests were statistically significant (p<0.0001) (S2 Table).

### Allometry

**Fig 5:**
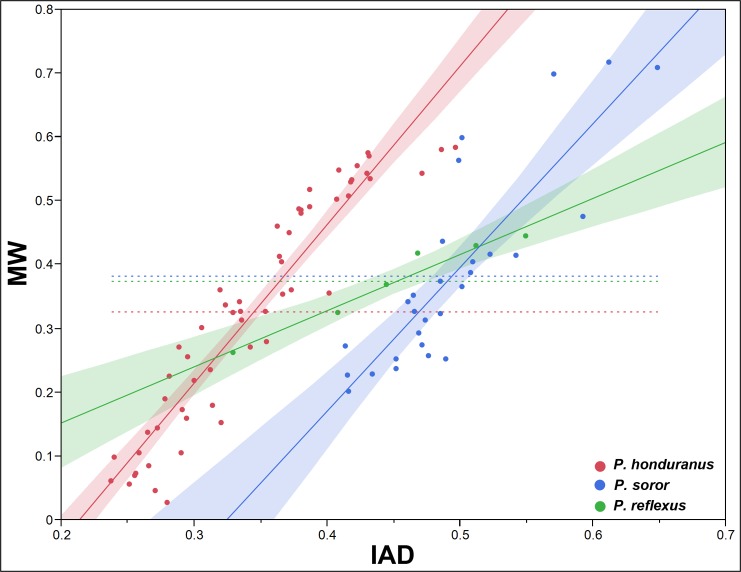
Allometric growth in the males of the genus *Platyneuromus*.

**Fig 6:**
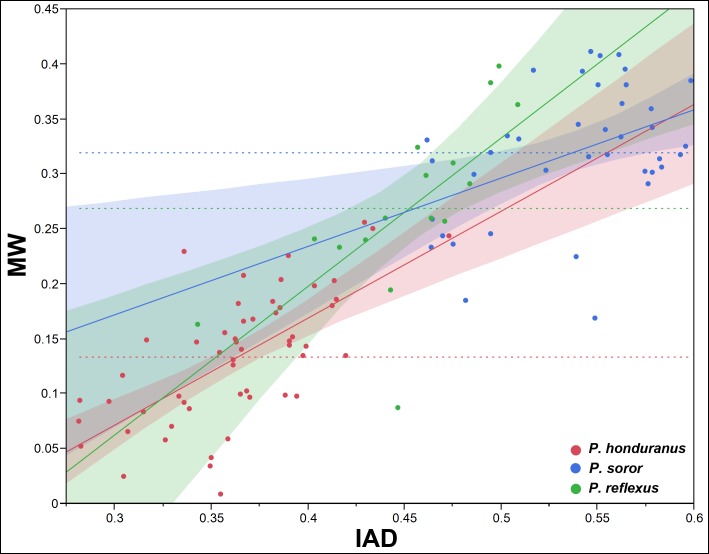
Allometric growth in the females of the genus *Platyneuromus*.

**Fig 7:**
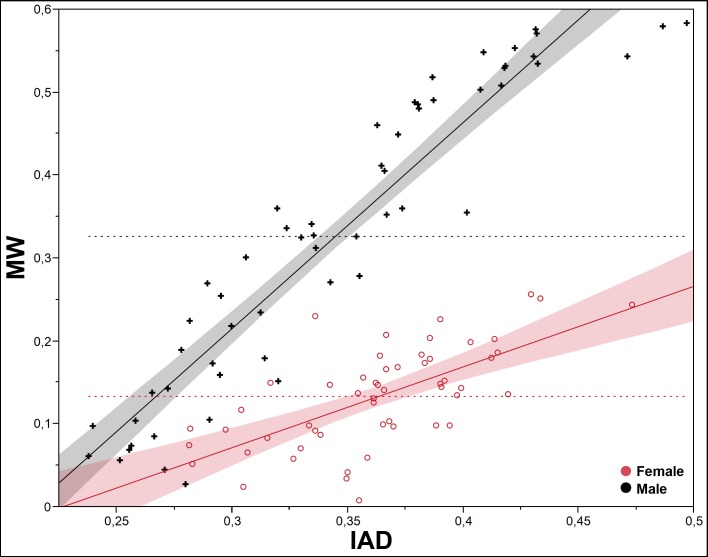
Allometric growth in *P*. *honduranus*. The developmental strategies correspond to a divergent growth trajectory. Linear fit male MW = -0.528809 + 2.4817574*IAD; female MW = -0.219867 + 0.9724537* IAD.

**Fig 8:**
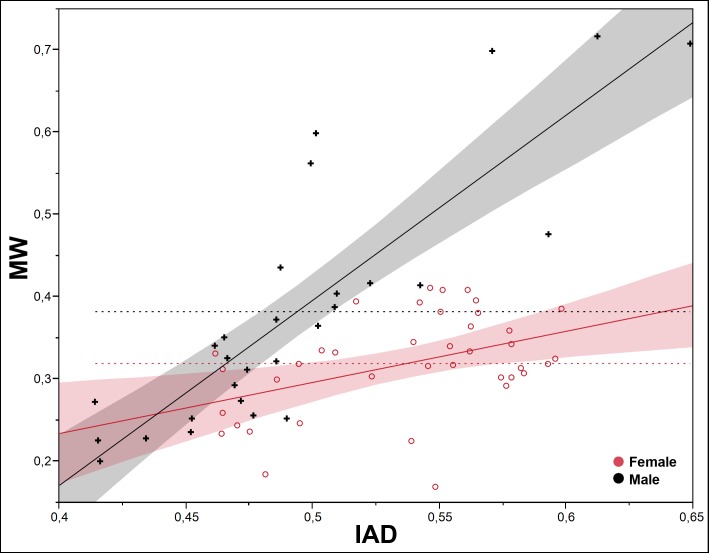
Allometric growth in *P*. *soror*. The developmental strategies correspond to a divergent growth trajectory. Linear fit male MW = -0.729184 + 2.2514065* IAD; female MW = -0.014324 + 0.6217066* IAD.

**Fig 9:**
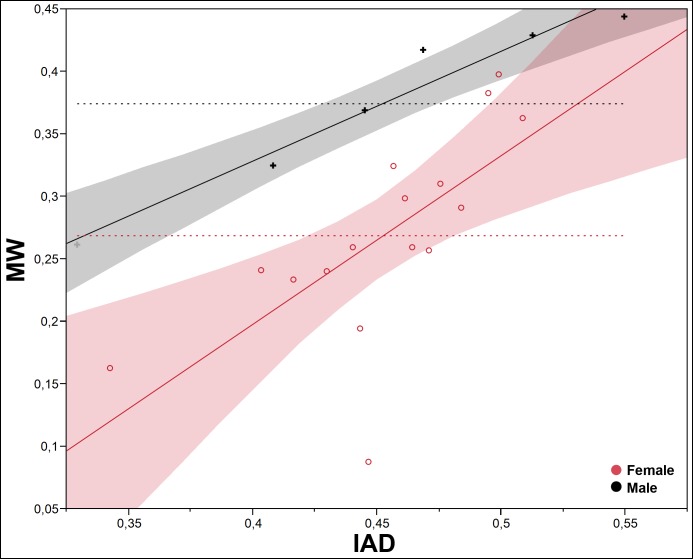
Allometric growth in *P*. *reflexus*. The developmental strategies correspond to a nearly parallel growth trajectory. Linear fit male MW = -0.023069 + 0.8791506* IAD; female MW = -0.34204 + 1.3501466* IAD.

To test the differential growth and allometry between the SMM and the POF, we use the interantennal distance (IAD) and the mesial width (MW), because they show one of the two lowest values of correlation between the two types of traits (0.5894, p<0.0001; Table 1). The pattern of differential growth between IAD and MW is very different interspecifically in both sexes (Figs 5 and 6); it is more evident in males (Fig 5), less marked in females (Fig 6). Intraspecifically, *P*. *honduranus* (Fig 7) and *P*. *soror* (Fig 8) show the largest differences and a divergent growth pattern, while *P*. *reflexus* is most similar in both sexes with an almost parallel growth pattern (Fig 9). The presence of positive allometry and the development of an oversized POF is clear in the males of *P*. *honduranus* and *P*. *soror* (Figs 7 and 8), while near isometry is present in the female of *P*. *honduranus* (Fig 7), and the female of *P*. *soror* exhibits negative allometry (Fig 8). There is positive allometry only in females of *P*. *reflexus*, so the growth rate expressed in the slope of the linear model is not very noticeable (Fig 9) (Table 1).

**Table 1:** Rate of allometric growth in the species of the genus *Platyneuromus*.

Species	Sex	Slope	Growth*	Correlation (R)	RSquared (R^2^)	Significance
*P*. *honduranus*	Male	2.4817	+ allometry	0.9389	0.8815	<0.0001*
	Female	0.9724	isometry	0.6642	0.4412	<0.0001*
*P*. *soror*	Male	2.2514	+ allometry	0.8536	0.7286	<0.0001*
	Female	0.6217	- allometry	0.4356	0.1897	<0.0056*
*P*. *reflexus*	Male	0.8791	- allometry	0.9775	0.9515	0.0009*
	Female	1.3501	+ isometry	0.6940	0.4816	0.0029*

* The allometric growth was calculated using the interocular distance as the independent variable and the mesial width of POF as dependent variable.

### Sexual dimorphism

**Fig 10:**
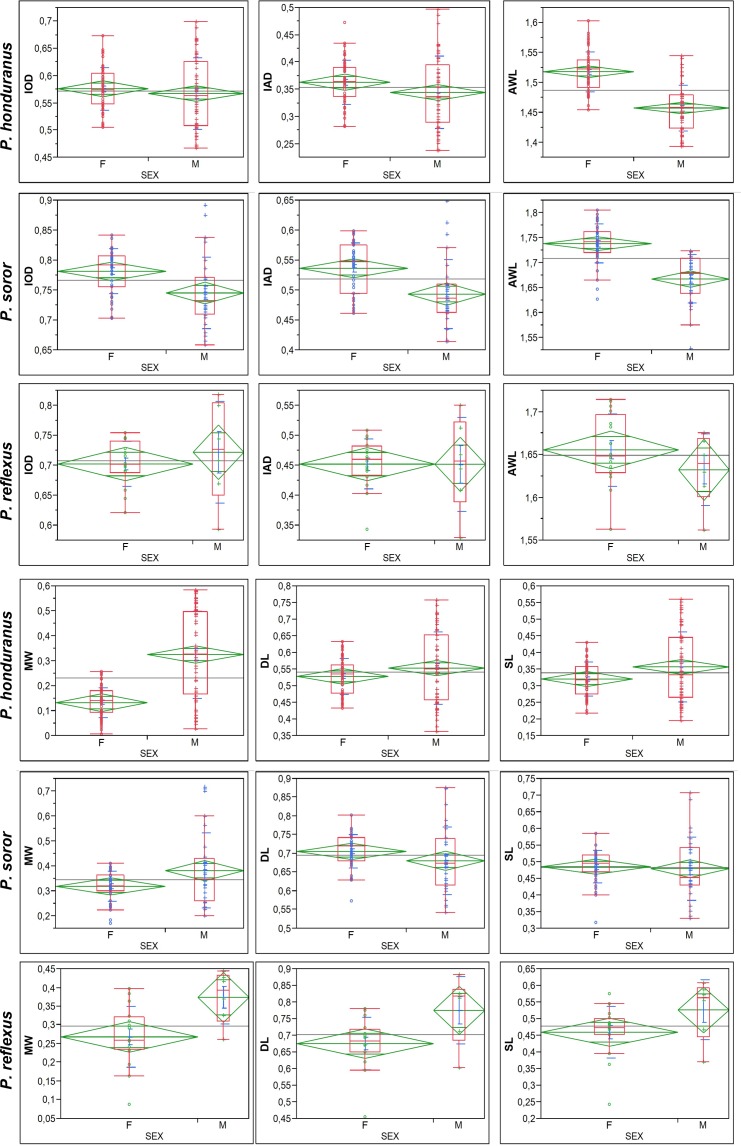
ANOVAs between all traits in each species with respect to sex. In the measures of body size there is a generally female-biased pattern of sexual size dimorphism, while in the POF measures, males are generally larger.

The SSD exhibits a female-biased scheme regarding SMM, with females in general larger than males (except *P*. *reflexus* for IOD), clearly seen in forewing length, while the males were clearly larger than females with respect to the POF, as seen in MW (except of DL and SL for *P*. *soror*) (Fig 10, S3 Table). The males of *P*. *honduranus*, the smallest males among the three species, present proportionally the biggest development of POF and the most remarkable sexual dimorphism.

### Relation between size and shape

**Fig 11:**
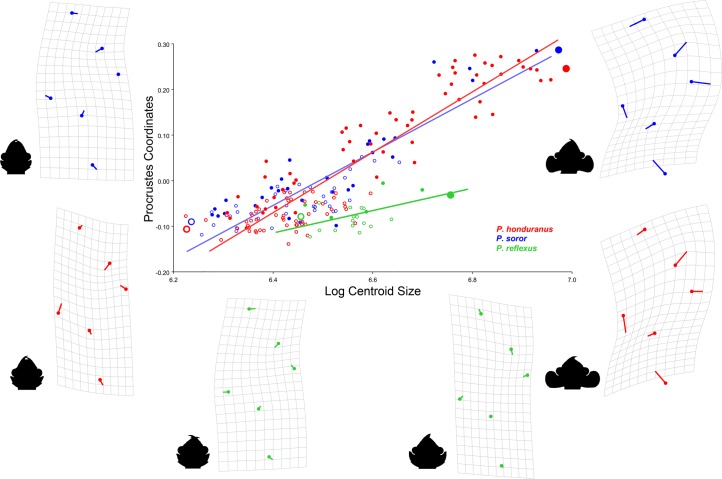
Geometric morphometric regression between centroid size and Procrustes coordinates. The lines show the fit of each species and the thin plate splines represent the Procrustes deformation of the POF from the smallest females (open circles) to the biggest males (closed circles). The POF is represented by six landmark coordinates.

The allometric correlation of the change of shape with respect to size shows different fit of the explained variance for the linear model in the genus, showing that the null hypothesis of independence of correlation of size with shape differed for each species (Fig 11). The proportion of variation as percentage for which the regression accounts the total variation was significantly higher in males, with greatest differences between *P*. *honduranus* and *P*. *soror* (over 70%) with respect to *P*. *reflexus* (44%) (Table 2). The allometric pattern among species can be seen as a scenario that represents all evolutionary changes in the three levels of allometry that are reciprocally interrelated; the character covariation among species (static), from different lineages that share a common ancestor (evolutionary), and in a single ontogenetic stage, the adult [50].

**Table 2:** Multivariate regressions of shape versus log-centroid size in *Platyneuromus*.

			Sum of squares of Procrustes coordinates			
Species	Sex	% explained variance	Total	Predicted	Residual	P-value
*P*. *honduranus*	Male	71.9244	0.965049	0.694105	0.270943	<0.0001
	Female	8.2683	0.178132	0.014728	0.163404	0.0011
*P*. *soror*	Male	71.3105	0.488285	0.348198	0.140086	<0.0001
	Female	25.2988	0.192777	0.048770	0.144007	<0.0001
*P*. *reflexus*	Male	44.4004	0.021783	0.009671	0.012111	0.0911
	Female	9.1807	0.036895	0.003387	0.033508	0.2190
GENERAL		54.29	3.389062	1.840226	1.548835	<0.0001

## Discussion

Different models of selective forces have been proposed and tested to explain allometry within a framework of natural or sexual selection, with a link between positive allometry and sexual selection generally accepted when exaggerated traits are present [7]. However, it has recently been proposed that cranial structures (ornaments) might play a determinant role in giantism along lineages in evolutionary time in theropod dinosaurs [29], therefore the ornament being a possible cause of change and not a result of selection, as usually has been described. It has also been proposed that increased complexity of exaggerated structures might not always be linked to sexual selection processes, but be a by-product of an increase in body size in a lineage along evolutionary time [28]. Nonetheless, with scaling exponents so high and the presence of contrasting SSD in *Platyneuromus*, sexual selection would certainly be the subjacent force that drives the evolution of the POF, as previously advanced in other studies [5,6]. We base this hypothesis in that insects (specifically holometabolous) do not continue to grow after reaching the adult stage (unlike vertebrates); also, in *Platyneuromus*, the species with the highest proportionate development and deformation of POF (*P*. *honduranus*) is not the one with the largest individuals, and a species with relatively large individuals (*P*. *reflexus*), has the lowest deformation of the POF.

Although scarce, observational evidence favors this conclusion. Contreras-Ramos [40] observed a discrete precopulatory courtship that included a face-to-face intimidating behavior in males. Similar displays have been described for other insects with cephalic expansions [51], so the POF might perform functions of visual display between males or be directed to females, a common phenomenon that involves structures with greatest external visibility and responsiveness to sexual selection (v.g. [33]). On the other hand, exaggerated allometric structures under a natural selection scenario may deploy functional roles as locomotion, thermoregulation, defense, species recognition, specialized coloniality, and others [11,12], and none of these mechanisms seems feasible for the POF in *Platyneuromus*. Instead, at least in *P*. *soror*, a strong odor has been noticed in males, which may involve pheromone communication between sexes [40].

The results show clear interspecific differences in SSD and allometric scaling of the POF in *Platyneuromus*. In two species with a clear and high allometric scaling (*P*. *honduranus* and *P*. *soror*), the POF is also notably different in shape: females have the POF semi triangular (also in males with slight growth of POF), but males with a bigger POF have it rounded, and in *P*. *reflexus*, the species with a non-positive allometry in POF, smaller males are similar in size and shape to females, whereas the bigger males only differ in size, with the shape similar to the females.

These differences in size and/or shape may be related to the mode and intensity of courtship behavior, but this aspect has not been studied in detail. Also, a phylogenetic framework to analyze the evolution of mating behavior and the divergence of size and shape of the POF remains as an attractive field to explore. Within a general pattern of sexual dimorphism in the animal kingdom, the female has the null or most moderate expression of the sexually selected trait, which corresponds with the plesiomorphic condition. Interestingly, in a phylogenetic study in *Corydalus*, another dobsonfly genus with cephalic exaggerated traits, Contreras-Ramos [38] found that some phylogenetically basal species were monomorphic.

The fact that these species are mutually ornamented, unlike other genera of dobsonflies with exaggerated traits (v.g. *Corydalus*), supports the presence of a POF as an ancestral trait (v.g. a genus synapomorphy), however a differential degree of development (higher in males), highlights the possible occurrence of mechanisms of selection that operate distinctly between sexes. Causes that promote this condition may be a response to a mutual sexual selection, where the traditional binary Darwinian model of sexually selected traits, weapon *vs* ornament, is insufficient. In fact, both models could be interdependent [25,52]; ornaments may be displayed in intersexual rivalry and male weapons can be seen as a signal of fitness by the mate. However, the most frequent condition of female ornamentation in a sexual context is that both sexes develop similar phenotypes. Yet, this is not the case, as females of the three species have the smallest POF. It has been documented that the female uses the ornamental trait in competition for non-sexual resources, in an ecological context, such as to obtain food for reproduction or offspring [53], however this has not been tested in *Platyneuromus*. If discovered, it may indicate an ancestral function before sexual selection played the leading role for highest development of the POF in males.

The allometric trajectories between species and sexes show two different developmental strategies: *P*. *honduranus* and *P*. *soror* fit with a divergent intersexual growth model, whereas *P*. *reflexus* exhibits a nearly parallel trajectory. In the first case, different developmental processes in sex-specific regulation of growth may be acting, in the second, similar processes regulate growth in both sexes [13]. These significant differences in the allometry between closely related species might indicate an evolutionary process of the scaling relationships, which is determining the rate of size and shape differentiation in the POF.

The different expressions of the POF are difficult to elucidate because several developmental phenomena are involved, such as different rates of allometric scale with distinct growth patterns, SSD, as well as the intra and interspecific size and shape variation of POF. Yet, a dual ornamentation suggests that the mechanisms of development of POF are derived from multi-causal effects, where the divergence time of speciation, sexual behavior, as well as different models of evolution in different proportion in the three species are involved. Local polymorphism in size, as well as other population level phenomena, such as differential food resource allocation in larvae, make explanations of this phenomenon more complex.

Empirical behavioral data and a phylogenetic framework should shed light to explain the direction and rate of the change in size and shape of the POF. A correlation with courtship behavior and intensity of sexual competition has been done in studies with other insects [31,54,55]. These next steps will help to explain comprehensively the origin and evolution of this biological form, and contribute to one of the most interesting and challenging contemporary research areas in the evo-devo field.

## Conclusions

This study supports an evolutionary interpretation of the growth rate of the postocular flange in *Platyneuromus* under sexual selection over other evolutionary forces, because the largest positive allometry is present in males of the species with the highest sexual dimorphism. The main premise is that this structure, with a great positive allometry, increases the success of mating.

We are aware that not only sexual selection is the possible path to the evolution of an ornamental-weaponry trait as the POF, however we contend is the best interpretation on the basis of the available data. And this is because of the extreme intra and interspecific differences in the allometric scale and sexual dimorphism. Until empirical behavioral and phylogenetic studies are performed, a more complete explanation may become available. The present study, with three species of a single dobsonfly genus, with distinct expressions of sexual dimorphism and allometric growth, offers an opportunity to explore the basis of the origin and evolution of phenotypic traits and their sexual divergence, clarifying the contribution of selective forces sustained in sexual behavioral strategies.

## Supporting information

S1 Table Specimen measurements log-transformed (IOD = interocular distance, IAD = interantennal distance, AWL = anterior wing length, MW = mesial width, DL = diagonal length, SL = postocular spine length; M = male, F = female). (DOCX)

S2 Table Pairwise correlations between measures of two types of traits (Body, indicative of a standard body measure: IOD = interocular distance, IAD = interantennal distance, AWL = anterior wing length; POF, indicative of a post ocular flange measure: MW = mesial width, DL = diagonal length, SL = postocular spine length; Signif. Prob. = significance of probability). (DOCX)

S3 Table Means for one-way ANOVA (IOD = interocular distance, IAD = interantennal distance, AWL = anterior wing length, MW = mesial width, DL = diagonal length, SL = postocular spine length; F = female, M = male, *n* = number of specimens). (DOCX)
