# Supplementary material for: The nature of allometry in an exaggerated trait: The postocular flange in Platyneuromus Weele (Insecta: Megaloptera)
Source: PLoS One. 2017 Feb 17;12(2):e0172388. doi: 10.1371/journal.pone.0172388 (PMC5315299; doi:10.1371/journal.pone.0172388)
Supplement: S1 Table — (DOCX) [file pone.0172388.s001.docx]

**S1 Table.** Specimen measurements log-transformed (IOD = interocular distance, IAD = interantennal distance, AWL = anterior wing length, MW = mesial width, DL = diagonal length, SL = postocular spine length; M = male, F = female).

| **Species** | **Sex** | **IOD** | **IAD** | **AWL** | **MW** | **DL** | **SL** |
| --- | --- | --- | --- | --- | --- | --- | --- |
| *P. honduranus* | M | 0.5039268 | 0.29136885 | 1.42258984 | 0.173058 | 0.45065703 | 0.22098103 |
| *P. honduranus* | M | 0.50447086 | 0.27783833 | 1.40925665 | 0.1892741 | 0.4771806 | 0.24536459 |
| *P. honduranus* | M | 0.46996921 | 0.25527251 | 1.42258984 | 0.06941277 | 0.36162397 | 0.19563125 |
| *P. honduranus* | M | 0.62746827 | 0.41664051 | 1.48129927 | 0.50798803 | 0.67112747 | 0.4940293 |
| *P. honduranus* | M | 0.53554728 | 0.30578115 | 1.45682135 | 0.30119934 | 0.54044085 | 0.34209486 |
| *P. honduranus* | M | 0.53932706 | 0.31993844 | 1.43264866 | 0.15154145 | 0.47522941 | 0.26159114 |
| *P. honduranus* | M | 0.69940408 | 0.48643048 | 1.54481191 | 0.5798236 | 0.75789837 | 0.56078868 |
| *P. honduranus* | M | 0.58647478 | 0.36660971 | 1.47363293 | 0.35324476 | 0.55562641 | 0.33222833 |
| *P. honduranus* | M | 0.59339684 | 0.37346372 | 1.46746011 | 0.36046655 | 0.5140441 | 0.32529805 |
| *P. honduranus* | M | 0.50283664 | 0.26599637 | 1.41094586 | 0.08477997 | 0.43103712 | 0.2268653 |
| *P. honduranus* | M | 0.51281776 | 0.28148789 | 1.44994099 | 0.22456932 | 0.42631603 | 0.28257 |
| *P. honduranus* | M | 0.61394748 | 0.3870337 | 1.4740705 | 0.49060452 | 0.65360706 | 0.43684299 |
| *P. honduranus* | M | 0.55726653 | 0.3193143 | 1.45301239 | 0.35975772 | 0.5558704 | 0.34466927 |
| *P. honduranus* | M | 0.50582803 | 0.29003461 | 1.41962536 | 0.1049512 | 0.46086478 | 0.27573738 |
| *P. honduranus* | M | 0.60151678 | 0.364551 | 1.46538285 | 0.41188895 | 0.60791695 | 0.3767904 |
| *P. honduranus* | M | 0.62314588 | 0.380573 | 1.48101242 | 0.48039792 | 0.64042578 | 0.43155031 |
| *P. honduranus* | M | 0.63558427 | 0.38667728 | 1.47813343 | 0.51823353 | 0.65237593 | 0.41270409 |
| *P. honduranus* | M | 0.66885165 | 0.42242568 | 1.51174971 | 0.55391699 | 0.70321139 | 0.5413071 |
| *P. honduranus* | M | 0.64048144 | 0.41796964 | 1.49927458 | 0.53008951 | 0.69771569 | 0.4833833 |
| *P. honduranus* | M | 0.57065967 | 0.33605928 | 1.44916973 | 0.31284141 | 0.56178927 | 0.36873118 |
| *P. honduranus* | M | 0.54369563 | 0.3232521 | 1.46059719 | 0.33638568 | 0.53517255 | 0.35601438 |
| *P. honduranus* | M | 0.5344069 | 0.31196566 | 1.44279323 | 0.23426159 | 0.46462813 | 0.28974276 |
| *P. honduranus* | M | 0.48287358 | 0.25139485 | 1.39950066 | 0.05642458 | 0.39797301 | 0.23265565 |
| *P. honduranus* | M | 0.47304881 | 0.23954972 | 1.39322412 | 0.09755923 | 0.42927575 | 0.26856393 |
| *P. honduranus* | M | 0.49387611 | 0.27966694 | 1.39828731 | 0.02740262 | 0.44804897 | 0.26047924 |
| *P. honduranus* | M | 0.49331861 | 0.27207379 | 1.42553422 | 0.14305542 | 0.43362265 | 0.25580781 |
| *P. honduranus* | M | 0.63958609 | 0.40857913 | 1.47596159 | 0.54849383 | 0.66728107 | 0.50106474 |
| *P. honduranus* | M | 0.58557352 | 0.3628593 | 1.47986311 | 0.46005699 | 0.61958477 | 0.38982146 |
| *P. honduranus* | M | 0.64424159 | 0.41846702 | 1.4984484 | 0.53265121 | 0.68424016 | 0.44556217 |
| *P. honduranus* | M | 0.48967729 | 0.27044591 | 1.40294883 | 0.04514688 | 0.41246391 | 0.23647932 |
| *P. honduranus* | M | 0.65705585 | 0.43200669 | 1.52504481 | 0.57071686 | 0.71998886 | 0.53493565 |
| *P. honduranus* | M | 0.59339684 | 0.36586222 | 1.47769993 | 0.40481833 | 0.57097818 | 0.38456486 |
| *P. honduranus* | M | 0.53428001 | 0.29972515 | 1.4372748 | 0.21874026 | 0.48944236 | 0.31275476 |
| *P. honduranus* | M | 0.59769519 | 0.38039216 | 1.46923274 | 0.48584205 | 0.65198704 | 0.44234178 |
| *P. honduranus* | M | 0.57345182 | 0.35372394 | 1.45833563 | 0.32578445 | 0.54324334 | 0.30769709 |
| *P. honduranus* | M | 0.68779641 | 0.49665294 | 1.53995384 | 0.58336996 | 0.7434102 | 0.55139632 |
| *P. honduranus* | M | 0.66332393 | 0.43248826 | 1.51851394 | 0.53504588 | 0.69242211 | 0.52072976 |
| *P. honduranus* | M | 0.63508144 | 0.40722089 | 1.48158594 | 0.5028421 | 0.66836541 | 0.48475525 |
| *P. honduranus* | M | 0.52100725 | 0.28891961 | 1.44513697 | 0.26992136 | 0.49104637 | 0.28124478 |
| *P. honduranus* | M | 0.66651798 | 0.43120289 | 1.51295108 | 0.57553537 | 0.71650166 | 0.52972928 |
| *P. honduranus* | M | 0.48756256 | 0.25815819 | 1.41279643 | 0.10479088 | 0.41987648 | 0.24294374 |
| *P. honduranus* | M | 0.50542133 | 0.26505379 | 1.44200916 | 0.13776858 | 0.42656006 | 0.22022842 |
| *P. honduranus* | M | 0.68859778 | 0.47129171 | 1.51108085 | 0.54340742 | 0.71401273 | 0.49511538 |
| *P. honduranus* | M | 0.46612587 | 0.23754374 | 1.42340973 | 0.06104514 | 0.42090092 | 0.23436541 |
| *P. honduranus* | M | 0.66520563 | 0.43039759 | 1.53007157 | 0.54350931 | 0.71671184 | 0.49356957 |
| *P. honduranus* | M | 0.5728716 | 0.34222523 | 1.45999526 | 0.27067651 | 0.54960766 | 0.32480289 |
| *P. honduranus* | M | 0.57909733 | 0.37180646 | 1.47639683 | 0.44982847 | 0.60920583 | 0.45025219 |
| *P. honduranus* | M | 0.47041049 | 0.25623653 | 1.39619935 | 0.07343239 | 0.37842879 | 0.20704432 |
| *P. honduranus* | M | 0.51041095 | 0.29490691 | 1.41680687 | 0.25499495 | 0.45208712 | 0.24899034 |
| *P. honduranus* | M | 0.53605316 | 0.32960125 | 1.42422807 | 0.32550951 | 0.51252564 | 0.2924333 |
| *P. honduranus* | M | 0.5470359 | 0.33425264 | 1.4407517 | 0.34192098 | 0.57930332 | 0.3973855 |
| *P. honduranus* | M | 0.55120594 | 0.33505652 | 1.45999526 | 0.32725674 | 0.56018889 | 0.32735076 |
| *P. honduranus* | M | 0.51308436 | 0.29468662 | 1.42406453 | 0.15934157 | 0.46664375 | 0.27154423 |
| *P. honduranus* | M | 0.59350759 | 0.37912415 | 1.47697647 | 0.48768408 | 0.61906619 | 0.43336815 |
| *P. honduranus* | M | 0.57518785 | 0.40157285 | 1.46657107 | 0.35467688 | 0.59070589 | 0.37893362 |
| *P. honduranus* | M | 0.53032779 | 0.31386722 | 1.43743344 | 0.17966093 | 0.47647802 | 0.31315617 |
| *P. honduranus* | M | 0.56241183 | 0.35487642 | 1.44994099 | 0.27839001 | 0.51514765 | 0.30046939 |
| *P. soror* | M | 0.7084209 | 0.48543748 | 1.62190296 | 0.32277112 | 0.64700133 | 0.46181055 |
| *P. soror* | M | 0.66511174 | 0.41597441 | 1.5748412 | 0.20064534 | 0.54201831 | 0.33708171 |
| *P. soror* | M | 0.8917604 | 0.64875021 | 1.71382642 | 0.70870807 | 0.87280985 | 0.60309672 |
| *P. soror* | M | 0.77136697 | 0.50947135 | 1.6707096 | 0.40390943 | 0.69341122 | 0.52855035 |
| *P. soror* | M | 0.73774907 | 0.48699689 | 1.63758979 | 0.43656653 | 0.76689744 | 0.53706693 |
| *P. soror* | M | 0.78554337 | 0.52244423 | 1.6735738 | 0.41687174 | 0.72472223 | 0.50141395 |
| *P. soror* | M | 0.79982283 | 0.54220278 | 1.69037331 | 0.41450372 | 0.73199628 | 0.54488499 |
| *P. soror* | M | 0.71933129 | 0.48967729 | 1.61394748 | 0.25212212 | 0.57502495 | 0.3519489 |
| *P. soror* | M | 0.71982829 | 0.47654181 | 1.69311112 | 0.25634967 | 0.62926277 | 0.43344179 |
| *P. soror* | M | 0.75273969 | 0.49899936 | 1.61752454 | 0.5628755 | 0.74208696 | 0.55524564 |
| *P. soror* | M | 0.74717867 | 0.47421626 | 1.64473393 | 0.31240376 | 0.69507872 | 0.43395215 |
| *P. soror* | M | 0.72468539 | 0.46612587 | 1.60627385 | 0.32620678 | 0.67770913 | 0.43169499 |
| *P. soror* | M | 0.83739902 | 0.59284268 | 1.68672562 | 0.47598917 | 0.78879672 | 0.57120089 |
| *P. soror* | M | 0.76760107 | 0.50133318 | 1.64137495 | 0.59911857 | 0.77541382 | 0.58988484 |
| *P. soror* | M | 0.6731131 | 0.41530729 | 1.67430989 | 0.22630082 | 0.56315346 | 0.36686357 |
| *P. soror* | M | 0.71340653 | 0.46119829 | 1.71733758 | 0.34146618 | 0.61955244 | 0.44250172 |
| *P. soror* | M | 0.71742084 | 0.47143841 | 1.69810055 | 0.27417402 | 0.60077257 | 0.43581889 |
| *P. soror* | M | 0.67960957 | 0.45224658 | 1.70492229 | 0.25197628 | 0.62819166 | 0.42781544 |
| *P. soror* | M | 0.74296067 | 0.48557948 | 1.72255166 | 0.37337733 | 0.67069476 | 0.49087529 |
| *P. soror* | M | 0.70415052 | 0.46923274 | 1.69337515 | 0.29306408 | 0.63393886 | 0.44390417 |
| *P. soror* | M | 0.8753507 | 0.61225391 | 1.71037126 | 0.71655839 | 0.87560582 | 0.70675362 |
| *P. soror* | M | 0.8309734 | 0.57077637 | 1.69949085 | 0.69906901 | 0.83044495 | 0.68761546 |
| *P. soror* | M | 0.65877432 | 0.41413736 | 1.52878819 | 0.27306844 | 0.55722079 | 0.33052335 |
| *P. soror* | M | 0.72230479 | 0.46508529 | 1.66200188 | 0.35189869 | 0.6612304 | 0.44348516 |
| *P. soror* | M | 0.76804581 | 0.50852972 | 1.65176245 | 0.38796791 | 0.67758141 | 0.4750869 |
| *P. soror* | M | 0.69284692 | 0.43408964 | 1.71416205 | 0.22828997 | 0.60691316 | 0.42573958 |
| *P. soror* | M | 0.75762375 | 0.50201722 | 1.72115084 | 0.36504785 | 0.67386838 | 0.48199124 |
| *P. soror* | M | 0.71323846 | 0.45178644 | 1.70884564 | 0.23654482 | 0.6142379 | 0.40575775 |
| *P. reflexus* | M | 0.7994784 | 0.51241755 | 1.6751365 | 0.42881487 | 0.82313631 | 0.5873708 |
| *P. reflexus* | M | 0.59262082 | 0.32919442 | 1.56169753 | 0.26196431 | 0.60202308 | 0.3705945 |
| *P. reflexus* | M | 0.74491854 | 0.46849503 | 1.64923747 | 0.41728232 | 0.82398778 | 0.57205566 |
| *P. reflexus* | M | 0.66968871 | 0.40790054 | 1.61341895 | 0.32486867 | 0.71132798 | 0.46822175 |
| *P. reflexus* | M | 0.81842386 | 0.54949371 | 1.66604974 | 0.44407916 | 0.882755 | 0.60833024 |
| *P. reflexus* | M | 0.70850588 | 0.44498111 | 1.62991904 | 0.36925854 | 0.81228752 | 0.55538831 |
| *P. honduranus* | F | 0.64394591 | 0.33605928 | 1.56608378 | 0.22918764 | 0.60799514 | 0.40480123 |
| *P. honduranus* | F | 0.58759873 | 0.39915433 | 1.52737208 | 0.14305854 | 0.55876615 | 0.29615602 |
| *P. honduranus* | F | 0.57298771 | 0.36660971 | 1.53160663 | 0.16552624 | 0.54225014 | 0.33588496 |
| *P. honduranus* | F | 0.58793535 | 0.3900515 | 1.53769319 | 0.22564005 | 0.55225075 | 0.37042611 |
| *P. honduranus* | F | 0.5289167 | 0.3047059 | 1.47885497 | 0.02424752 | 0.43186533 | 0.22605057 |
| *P. honduranus* | F | 0.5653755 | 0.36398783 | 1.50310944 | 0.18211779 | 0.47370151 | 0.25689566 |
| *P. honduranus* | F | 0.53882499 | 0.32960125 | 1.49734438 | 0.06997133 | 0.45395555 | 0.26955497 |
| *P. honduranus* | F | 0.50906805 | 0.28148789 | 1.46119829 | 0.07464459 | 0.47241414 | 0.28873871 |
| *P. honduranus* | F | 0.59295357 | 0.3834563 | 1.53249959 | 0.17294428 | 0.55398249 | 0.35588234 |
| *P. honduranus* | F | 0.57194164 | 0.37014285 | 1.48187241 | 0.09622851 | 0.5394387 | 0.34019418 |
| *P. honduranus* | F | 0.54481191 | 0.33845649 | 1.48401496 | 0.08634915 | 0.48220591 | 0.28643626 |
| *P. honduranus* | F | 0.54294985 | 0.3165993 | 1.45362407 | 0.14886784 | 0.50278888 | 0.31358041 |
| *P. honduranus* | F | 0.59703667 | 0.39445168 | 1.52465571 | 0.09762861 | 0.53541566 | 0.31478754 |
| *P. honduranus* | F | 0.57217431 | 0.361161 | 1.52387648 | 0.12576745 | 0.52613242 | 0.3088311 |
| *P. honduranus* | F | 0.6735738 | 0.47319491 | 1.60346916 | 0.24368033 | 0.63370069 | 0.43049108 |
| *P. honduranus* | F | 0.61658053 | 0.41946007 | 1.53706314 | 0.13497672 | 0.54962237 | 0.34810228 |
| *P. honduranus* | F | 0.57989787 | 0.3569814 | 1.53466058 | 0.15525403 | 0.5391376 | 0.32725469 |
| *P. honduranus* | F | 0.6097011 | 0.39741854 | 1.52814508 | 0.13483666 | 0.59116816 | 0.36733542 |
| *P. honduranus* | F | 0.60659631 | 0.38560627 | 1.56525734 | 0.17817665 | 0.59411177 | 0.4104381 |
| *P. honduranus* | F | 0.57065967 | 0.36828689 | 1.47842219 | 0.10238225 | 0.50558812 | 0.31485275 |
| *P. honduranus* | F | 0.60325266 | 0.39216915 | 1.57030939 | 0.15155064 | 0.55104968 | 0.3527689 |
| *P. honduranus* | F | 0.50569251 | 0.28194193 | 1.50879897 | 0.09359327 | 0.47038844 | 0.25252294 |
| *P. honduranus* | F | 0.64777405 | 0.43360984 | 1.5824043 | 0.25042732 | 0.61975158 | 0.38763183 |
| *P. honduranus* | F | 0.64048144 | 0.42942926 | 1.57368369 | 0.25623653 | 0.61454184 | 0.38933091 |
| *P. honduranus* | F | 0.5728716 | 0.3588862 | 1.50745106 | 0.05918462 | 0.47458045 | 0.26833221 |
| *P. honduranus* | F | 0.59824319 | 0.36586222 | 1.55157197 | 0.14028487 | 0.56840122 | 0.36234862 |
| *P. honduranus* | F | 0.63387226 | 0.413635 | 1.57783634 | 0.20238477 | 0.63305113 | 0.40409273 |
| *P. honduranus* | F | 0.60508946 | 0.38201704 | 1.54182877 | 0.1835403 | 0.59084518 | 0.37595975 |
| *P. honduranus* | F | 0.58308537 | 0.36698298 | 1.54629584 | 0.20726265 | 0.55365126 | 0.35001336 |
| *P. honduranus* | F | 0.56784945 | 0.35506821 | 1.52192225 | 0.00810173 | 0.46188853 | 0.26250092 |
| *P. honduranus* | F | 0.54888056 | 0.34986008 | 1.49789674 | 0.04170448 | 0.52305937 | 0.30065634 |
| *P. honduranus* | F | 0.54789772 | 0.32654067 | 1.47899913 | 0.05744928 | 0.47829805 | 0.25482109 |
| *P. honduranus* | F | 0.5133508 | 0.29710365 | 1.47813343 | 0.09274169 | 0.46050094 | 0.24992687 |
| *P. honduranus* | F | 0.58149454 | 0.37180646 | 1.51295108 | 0.16792256 | 0.55991739 | 0.34643513 |
| *P. honduranus* | F | 0.59681694 | 0.38596357 | 1.52153034 | 0.20338649 | 0.55593561 | 0.34230817 |
| *P. honduranus* | F | 0.58546073 | 0.3630476 | 1.52322604 | 0.14673253 | 0.4481016 | 0.21834116 |
| *P. honduranus* | F | 0.55642312 | 0.34966598 | 1.47610672 | 0.03420318 | 0.50517305 | 0.3032868 |
| *P. honduranus* | F | 0.57576498 | 0.361161 | 1.50555694 | 0.13033377 | 0.53170367 | 0.31042777 |
| *P. honduranus* | F | 0.59039595 | 0.38845645 | 1.52048353 | 0.0982248 | 0.53612014 | 0.33471706 |
| *P. honduranus* | F | 0.56359973 | 0.34262004 | 1.48244479 | 0.14684093 | 0.51555595 | 0.28960679 |
| *P. honduranus* | F | 0.60659631 | 0.39058188 | 1.51732788 | 0.14396047 | 0.53000365 | 0.31683597 |
| *P. honduranus* | F | 0.61246596 | 0.39040516 | 1.54654266 | 0.14825088 | 0.50994946 | 0.27510165 |
| *P. honduranus* | F | 0.63437649 | 0.41497335 | 1.53249959 | 0.18565546 | 0.5926652 | 0.38802123 |
| *P. honduranus* | F | 0.53122338 | 0.31513032 | 1.50064806 | 0.0832804 | 0.48857046 | 0.31427181 |
| *P. honduranus* | F | 0.61825735 | 0.41246055 | 1.56276854 | 0.17997095 | 0.57783405 | 0.3593706 |
| *P. honduranus* | F | 0.52452594 | 0.30405947 | 1.52439612 | 0.11649816 | 0.46100152 | 0.25144354 |
| *P. honduranus* | F | 0.50960571 | 0.28262211 | 1.47436198 | 0.05164637 | 0.44500605 | 0.26701207 |
| *P. honduranus* | F | 0.56761444 | 0.36530075 | 1.49954963 | 0.09919706 | 0.56660364 | 0.34525803 |
| *P. honduranus* | F | 0.55291145 | 0.3544926 | 1.52153034 | 0.13701211 | 0.54312025 | 0.35291538 |
| *P. honduranus* | F | 0.5939503 | 0.4034637 | 1.52621 | 0.19798313 | 0.58463987 | 0.37516595 |
| *P. honduranus* | F | 0.50866436 | 0.30685375 | 1.47392469 | 0.06525845 | 0.44091223 | 0.24410386 |
| *P. honduranus* | F | 0.5378191 | 0.33344727 | 1.49872397 | 0.09789214 | 0.54058722 | 0.34283309 |
| *P. honduranus* | F | 0.56749689 | 0.36248248 | 1.4881275 | 0.14928071 | 0.51683538 | 0.31723153 |
| *P. honduranus* | F | 0.53122338 | 0.33605928 | 1.49248101 | 0.09211333 | 0.47750037 | 0.27262124 |
| *P. soror* | F | 0.81304697 | 0.56253077 | 1.71307033 | 0.36404794 | 0.73395762 | 0.52469075 |
| *P. soror* | F | 0.79021477 | 0.54851226 | 1.71650416 | 0.16898042 | 0.69216455 | 0.45730801 |
| *P. soror* | F | 0.80002936 | 0.54629584 | 1.73965144 | 0.4111397 | 0.72103611 | 0.50262237 |
| *P. soror* | F | 0.73021684 | 0.46179856 | 1.6834073 | 0.33055987 | 0.66774425 | 0.44033594 |
| *P. soror* | F | 0.72664572 | 0.48158594 | 1.62716095 | 0.18466872 | 0.63892832 | 0.44753942 |
| *P. soror* | F | 0.791129 | 0.54567815 | 1.79000352 | 0.31582346 | 0.72055784 | 0.46286648 |
| *P. soror* | F | 0.78746048 | 0.55533633 | 1.80488875 | 0.31753685 | 0.72056115 | 0.50490155 |
| *P. soror* | F | 0.70286117 | 0.46419137 | 1.64601141 | 0.2333769 | 0.69794995 | 0.47367962 |
| *P. soror* | F | 0.73719264 | 0.47523522 | 1.72599326 | 0.23611132 | 0.5735643 | 0.31663073 |
| *P. soror* | F | 0.79035555 | 0.50906805 | 1.72288061 | 0.33187452 | 0.7277803 | 0.50682962 |
| *P. soror* | F | 0.80366192 | 0.57852461 | 1.73351825 | 0.34212845 | 0.69894308 | 0.48754843 |
| *P. soror* | F | 0.70372116 | 0.46463856 | 1.6652995 | 0.25906323 | 0.62826016 | 0.42078886 |
| *P. soror* | F | 0.80195224 | 0.55400432 | 1.6993173 | 0.34030131 | 0.75567138 | 0.5191846 |
| *P. soror* | F | 0.75503593 | 0.49471103 | 1.71028665 | 0.31881226 | 0.67512549 | 0.48193684 |
| *P. soror* | F | 0.82046419 | 0.59824319 | 1.75891189 | 0.38507515 | 0.74894119 | 0.53324444 |
| *P. soror* | F | 0.78936915 | 0.56217386 | 1.74397987 | 0.33394074 | 0.69112572 | 0.47302542 |
| *P. soror* | F | 0.80174662 | 0.55059521 | 1.76162719 | 0.3810609 | 0.69663418 | 0.47509271 |
| *P. soror* | F | 0.7044937 | 0.46448955 | 1.71949702 | 0.31152712 | 0.67079487 | 0.42721662 |
| *P. soror* | F | 0.83657728 | 0.57783634 | 1.74311763 | 0.35935351 | 0.74934512 | 0.50202952 |
| *P. soror* | F | 0.81796181 | 0.56110138 | 1.75004531 | 0.40868591 | 0.7629605 | 0.50544303 |
| *P. soror* | F | 0.79622731 | 0.55120594 | 1.75404239 | 0.40777319 | 0.76765669 | 0.5510106 |
| *P. soror* | F | 0.79183095 | 0.53995384 | 1.74421473 | 0.34522469 | 0.74367044 | 0.51904004 |
| *P. soror* | F | 0.8049568 | 0.54245195 | 1.782974 | 0.39309941 | 0.74232992 | 0.53350769 |
| *P. soror* | F | 0.78197067 | 0.51706387 | 1.71365852 | 0.3944114 | 0.72246363 | 0.50958152 |
| *P. soror* | F | 0.81895131 | 0.58331215 | 1.78268757 | 0.30647221 | 0.72094116 | 0.51793977 |
| *P. soror* | F | 0.76529593 | 0.52348633 | 1.76834206 | 0.30269017 | 0.68468382 | 0.47413173 |
| *P. soror* | F | 0.75800301 | 0.49485002 | 1.77092562 | 0.24575196 | 0.63326338 | 0.40714777 |
| *P. soror* | F | 0.73917663 | 0.48586333 | 1.75066265 | 0.29947679 | 0.70479204 | 0.48212292 |
| *P. soror* | F | 0.81050135 | 0.58206336 | 1.79685175 | 0.31340738 | 0.6871605 | 0.4835331 |
| *P. soror* | F | 0.77633791 | 0.5390761 | 1.78802688 | 0.2246936 | 0.68054309 | 0.48095216 |
| *P. soror* | F | 0.71750408 | 0.47011635 | 1.7420964 | 0.24351427 | 0.63007175 | 0.39954047 |
| *P. soror* | F | 0.79056663 | 0.56454771 | 1.78504496 | 0.395239 | 0.72460106 | 0.51531473 |
| *P. soror* | F | 0.81908308 | 0.59560644 | 1.76049788 | 0.32454583 | 0.72400948 | 0.52761444 |
| *P. soror* | F | 0.8417973 | 0.59284268 | 1.74538712 | 0.31775421 | 0.71078311 | 0.49533061 |
| *P. soror* | F | 0.80112919 | 0.56513915 | 1.73687436 | 0.38084774 | 0.80169314 | 0.58429843 |
| *P. soror* | F | 0.74491854 | 0.50351831 | 1.72468539 | 0.33448391 | 0.7483338 | 0.52440132 |
| *P. soror* | F | 0.80222624 | 0.57622614 | 1.73631681 | 0.29118897 | 0.68015324 | 0.46970275 |
| *P. soror* | F | 0.80733204 | 0.57863921 | 1.73399929 | 0.30146407 | 0.74383497 | 0.52103342 |
| *P. soror* | F | 0.81895131 | 0.57449427 | 1.73463984 | 0.30192806 | 0.72151467 | 0.49766346 |
| *P. reflexus* | F | 0.75396587 | 0.50893353 | 1.71256553 | 0.36277266 | 0.78101752 | 0.57436699 |
| *P. reflexus* | F | 0.69019608 | 0.44043677 | 1.64640373 | 0.2593119 | 0.69831372 | 0.47137827 |
| *P. reflexus* | F | 0.6993173 | 0.46419137 | 1.62797999 | 0.25949115 | 0.69839549 | 0.46796161 |
| *P. reflexus* | F | 0.68859778 | 0.44310646 | 1.6298172 | 0.19437279 | 0.61918206 | 0.39499421 |
| *P. reflexus* | F | 0.70286117 | 0.46149853 | 1.66275783 | 0.29844259 | 0.69306709 | 0.48454753 |
| *P. reflexus* | F | 0.64502907 | 0.4034637 | 1.60852603 | 0.24108056 | 0.6504241 | 0.45158228 |
| *P. reflexus* | F | 0.65782046 | 0.41647408 | 1.6242821 | 0.23309769 | 0.65041536 | 0.45322964 |
| *P. reflexus* | F | 0.74546517 | 0.49485002 | 1.7068031 | 0.3828362 | 0.75958702 | 0.51404145 |
| *P. reflexus* | F | 0.69688037 | 0.44653717 | 1.68241586 | 0.08714583 | 0.45409448 | 0.24172823 |
| *P. reflexus* | F | 0.68797462 | 0.43007506 | 1.63528264 | 0.24009725 | 0.67204796 | 0.45335045 |
| *P. reflexus* | F | 0.74460588 | 0.48387245 | 1.68618923 | 0.2912134 | 0.67071608 | 0.48326484 |
| *P. reflexus* | F | 0.72131588 | 0.47114497 | 1.64127576 | 0.25672977 | 0.72243236 | 0.50260736 |
| *P. reflexus* | F | 0.72802895 | 0.47538059 | 1.65040467 | 0.30980896 | 0.70557722 | 0.48771234 |
| *P. reflexus* | F | 0.71474876 | 0.45682135 | 1.70087671 | 0.32400038 | 0.67021059 | 0.47249023 |
| *P. reflexus* | F | 0.62096844 | 0.34281732 | 1.56312496 | 0.16282717 | 0.59507385 | 0.36342393 |
| *P. reflexus* | F | 0.74382322 | 0.49927458 | 1.71449741 | 0.39768631 | 0.77484165 | 0.54606986 |
