# Supplementary material for: The nature of allometry in an exaggerated trait: The postocular flange in Platyneuromus Weele (Insecta: Megaloptera)
Source: PLoS One. 2017 Feb 17;12(2):e0172388. doi: 10.1371/journal.pone.0172388 (PMC5315299; doi:10.1371/journal.pone.0172388)
Supplement: S2 Table — (DOCX) [file pone.0172388.s002.docx]

**S2 Table.** Pairwise correlations between measures of two types of traits (Body, indicative of a standard body measure: IOD = interocular distance, IAD = interantennal distance, AWL = anterior wing length; POF, indicative of a post ocular flange measure: MW = mesial width, DL = diagonal length, SL = postocular spine length; Signif. Prob. = significance of probability).

| **Variables** | **Type** | **Correlation** | **Lower 95%** | **Upper 95%** | **Signif. Prob.** |
| --- | --- | --- | --- | --- | --- |
| IAD x IOD | Body x body | 0.9833 | 0.9780 | 0.9874 | <0.0001 |
| SL x DL | POF x POF | 0.9783 | 0.9715 | 0.9836 | <0.0001 |
| AWL x IOD | Body x body | 0.9256 | 0.9028 | 0.9432 | <0.0001 |
| AWL x IAD | Body x body | 0.9065 | 0.8782 | 0.9285 | <0.0001 |
| DL x IOD | POF x body | 0.8957 | 0.8643 | 0.9201 | <0.0001 |
| DL x IAD | POF x body | 0.8815 | 0.8462 | 0.9091 | <0.0001 |
| SL x IOD | POF x body | 0.8726 | 0.8349 | 0.9021 | <0.0001 |
| SL x IAD | POF x body | 0.8595 | 0.8183 | 0.8919 | <0.0001 |
| SL x MW | POF x POF | 0.8088 | 0.7547 | 0.8519 | <0.0001 |
| DL x MW | POF x POF | 0.7835 | 0.7234 | 0.8318 | <0.0001 |
| DL x AWL | POF x body | 0.7495 | 0.6816 | 0.8046 | <0.0001 |
| SL x AWL | POF x body | 0.7216 | 0.6477 | 0.7821 | <0.0001 |
| MW x IOD | POF x body | 0.6031 | 0.5068 | 0.6846 | <0.0001 |
| MW x IAD | POF x body | 0.5894 | 0.4908 | 0.6731 | <0.0001 |
| MW x AWL | POF x body | 0.3537 | 0.2260 | 0.4694 | <0.0001 |
