# Supplementary material for: The nature of allometry in an exaggerated trait: The postocular flange in Platyneuromus Weele (Insecta: Megaloptera)
Source: PLoS One. 2017 Feb 17;12(2):e0172388. doi: 10.1371/journal.pone.0172388 (PMC5315299; doi:10.1371/journal.pone.0172388)
Supplement: S3 Table — (DOCX) [file pone.0172388.s003.docx]

**S3 table.** Means for one-way ANOVA (IOD = interocular distance, IAD = interantennal distance, AWL = anterior wing length, MW = mesial width, DL = diagonal length, SL = postocular spine length; F = female, M = male, *n* = number of specimens).

| **Species** | **Source** | **Sex** | ***n*** | **Means** | **Standard deviation** | **Standard error mean** | **Lower 95%** | **Upper 95%** |
| --- | --- | --- | --- | --- | --- | --- | --- | --- |
| *P. honduranus* | Sex by IOD | F | 54 | 0.576455 | 0.039158 | 0.00533 | 0.56577 | 0.58714 |
|  |  | M | 57 | 0.567673 | 0.065537 | 0.00868 | 0.55028 | 0.58506 |
| *P. soror* |  | F | 39 | 0.781818 | 0.037887 | 0.00607 | 0.76954 | 0.79410 |
|  |  | M | 28 | 0.745656 | 0.059345 | 0.01122 | 0.72264 | 0.76867 |
| *P. reflexus* |  | F | 16 | 0.702600 | 0.038013 | 0.00950 | 0.68234 | 0.72286 |
|  |  | M | 6 | 0.722273 | 0.084241 | 0.03439 | 0.63387 | 0.81068 |
| *P. honduranus* | Sex by IAD | F | 54 | 0.363427 | 0.040778 | 0.00555 | 0.35230 | 0.37456 |
|  |  | M | 57 | 0.344607 | 0.066225 | 0.00877 | 0.32703 | 0.36218 |
| *P. soror* |  | F | 39 | 0.536846 | 0.042546 | 0.00681 | 0.52305 | 0.55064 |
|  |  | M | 28 | 0.493739 | 0.057029 | 0.01078 | 0.47163 | 0.51585 |
| *P. reflexus* |  | F | 16 | 0.452430 | 0.041443 | 0.01036 | 0.43035 | 0.47451 |
|  |  | M | 6 | 0.452080 | 0.078106 | 0.03189 | 0.37011 | 0.53405 |
| *P. honduranus* | Sex by AWL | F | 54 | 1.51832 | 0.033563 | 0.00457 | 1.5092 | 1.5275 |
|  |  | M | 57 | 1.45781 | 0.038270 | 0.00507 | 1.4477 | 1.4680 |
| *P. soror* |  | F | 39 | 1.73864 | 0.038900 | 0.00623 | 1.7260 | 1.7513 |
|  |  | M | 28 | 1.66763 | 0.048430 | 0.00915 | 1.6489 | 1.6864 |
| *P. reflexus* |  | F | 16 | 1.65583 | 0.042424 | 0.01061 | 1.6332 | 1.6784 |
|  |  | M | 6 | 1.63258 | 0.041490 | 0.01694 | 1.5890 | 1.6761 |
| *P. honduranus* | Sex by MW | F | 54 | 0.133548 | 0.059699 | 0.00812 | 0.11725 | 0.14984 |
|  |  | M | 57 | 0.326421 | 0.175043 | 0.02319 | 0.27998 | 0.37287 |
| *P. soror* |  | F | 39 | 0.319437 | 0.060729 | 0.00972 | 0.29975 | 0.33912 |
|  |  | M | 28 | 0.382423 | 0.150420 | 0.02843 | 0.32410 | 0.44075 |
| *P. reflexus* |  | F | 16 | 0.268807 | 0.080628 | 0.02016 | 0.22584 | 0.31177 |
|  |  | M | 6 | 0.374378 | 0.070394 | 0.02874 | 0.30050 | 0.44825 |
| *P. honduranus* | Sex by DL | F | 54 | 0.529153 | 0.052945 | 0.00720 | 0.51470 | 0.54360 |
|  |  | M | 57 | 0.554072 | 0.109353 | 0.01448 | 0.52506 | 0.58309 |
| *P. soror* |  | F | 39 | 0.706270 | 0.045080 | 0.00722 | 0.69166 | 0.72088 |
|  |  | M | 28 | 0.681273 | 0.090844 | 0.01717 | 0.64605 | 0.71650 |
| *P. reflexus* |  | F | 16 | 0.675962 | 0.078533 | 0.01963 | 0.63411 | 0.71781 |
|  |  | M | 6 | 0.775920 | 0.101674 | 0.04151 | 0.66922 | 0.88262 |
| *P. honduranus* | Sex by SL | F | 54 | 0.321202 | 0.051178 | 0.00696 | 0.30723 | 0.33517 |
|  |  | M | 57 | 0.357499 | 0.105974 | 0.01404 | 0.32938 | 0.38562 |
| *P. soror* |  | F | 39 | 0.486071 | 0.047789 | 0.00765 | 0.47058 | 0.50156 |
|  |  | M | 28 | 0.480215 | 0.094721 | 0.01790 | 0.44349 | 0.51694 |
| *P. reflexus* |  | F | 16 | 0.460172 | 0.076959 | 0.01924 | 0.41916 | 0.50118 |
|  |  | M | 6 | 0.526994 | 0.90568 | 0.03697 | 0.43195 | 0.62204 |
